# Can proton therapy be considered a standard of care in oncology? Lessons from the United States

**DOI:** 10.1038/s41416-018-0324-2

**Published:** 2019-03-26

**Authors:** Anthony L. Zietman

**Affiliations:** Massachusetts General Hospital, Boston, MA USA

In April 2018, the New York Times ran an article documenting the exuberant overexpansion of proton beam therapy centres in the USA.^1^ Twenty-seven centres have opened, and a comparable number are in the planning stages; yet, over a third of these are in deep financial trouble, with mounting losses, debt restructuring, and bankruptcies. How did such a promising and exciting medical technology expand so far and then contract so fast? Was this due to problems in the technology itself, or aspects of healthcare financing unique to the USA? How did a technology expand so far ahead of its supportive evidence? And what are the implications for the UK as it stands on the threshold of its own proton age?

The radiation oncology field can be proud of having achieved many technological milestones on the road to developing a safe, accurate, and effective modality over the past century. Proton beam therapy stands at the point of this technological arrow, and to many practitioners it is nothing more than the natural next step from traditional photon beam radiotherapies. The big difference is that all prior radiotherapy developments were incremental, in everything from price to efficacy. Proton therapy, however, required a colossal investment in not only a piece of machinery but an entire facility, and on a scale never previously seen in medicine. Enthusiasts promised that the cost was justified in a technology that would change the entire game, and that downstream savings in terms of increased cure rates and reduced morbidity would pay back any investment. The theory was compelling, and the FDA gave approval in the 1980s on the assumption that few centres would, or could, be built. In the early days the focus was on paediatric cancers because the late effects of radiation in children can be devastating. Paediatric cancers are, however, rare, and so the technology was increasingly applied to more common oncologic scenarios such as prostate cancer.

## A contracting industry?

With the groundwork laid, very little happened for over a decade. The expansion of centres picked up speed from 2004 onwards, driven by a growing stream of prostate cancer patients detected with prostate-specific antigen (PSA), by a presumed willingness of insurers to pay, and by novel forms of debt financing that freed institutions from having to raise the capital for the centre themselves. The financial recession of 2009 slowed things a little but was not the cause of the current crisis; this relates to several factors. Firstly, many insurers are now declining to pay for proton treatment for, in some cases, anything other than paediatric cancers. This is because the evidence base did not keep up with the expansion of centres. Proton therapy costs substantially more to deliver than other sophisticated forms of radiation, yet insurers had no proof that they, and their patients, were getting value for money. Another New York Times headline in 2012 said it all; ‘It costs more, but is it worth more?’.^2^ A second problem was the contraction of the prostate cancer market. PSA screening, and thus detection rates, declined in the USA, and other technologically attractive alternative treatments emerged, such as robot-assisted prostatectomy and stereotactic body radiation therapy. The third problem was saturation of the market. With 27 proton beam therapy centres, there are simply not enough paying patients to go around, and gantries stand empty.

Despite aggressive direct-to-patient marketing campaigns and state-by-state lobbying to change the payment rules, proton therapy is no longer a sure-fire investment strategy. Those major academic centres who are philanthropically well endowed, or who purchased their facilities without debt, will cope and will survive. Many of the others will not survive, and we are thus at a ‘tipping point’.^3,7^ Payers agreeing to fund patients in trials, or at a reduced standard radiotherapy rate, will help some of the newer centres to survive. Single-gantry facilities and ‘miniaturisation’ will, in time, bring down the start-up costs.^5,6^ The proton bubble in the USA will not explode, but it will deflate,^4^ perhaps resetting to a degree of use commensurate with supportive evidence.

## Developing the evidence base

The significant failure of proton beam therapy in the USA has been its inability to develop the evidence base needed to ensure its survival. It is only in the last 5 years that a critical mass of willing proton centres has been reached to enable multi-centre studies, and it is only in the last 5 years that the urgency to conduct such trials has been felt. So far there has been a general reluctance to test proton therapy against more traditional radiation. Randomised trials in breast and lung cancer are accruing far below expectation, and it is only the multi-centre prostate cancer trial that looks set to complete in the near future. Other nations, such as Holland, have chosen to support the use of proton beam therapy on the basis of comparative planning for individual patients, rather than disease indications. If a proton plan for a patient spares more normal tissue from high doses of radiation than a conventional radiation plan, then, on theoretical grounds alone, that patient will be offered proton therapy. While it solves the payment conundrum, it does not help to develop the evidence base.

Thanks to its centrally funded healthcare system, the UK chose to introduce proton therapy following a national means assessment. The commission determined that one major facility in Manchester and another in London should serve the nation’s needs, based on current evidence and indications (primarily paediatric cancers, sarcomas, and tumours of the central nervous system). There will also be some capacity left over for trials and for an expansion of indications should the trials justify that. Some private centres are opening in parallel, which have an, as yet, undetermined ability to influence and destabilise the market, but, for now, we will set those aside.

With its robust trials infrastructure, the UK is well poised to perform the randomised studies that the USA, and indeed the rest of the world, have been incapable of conducting.^8^ The UK has proven itself capable of testing new technology as well as new drugs, and their trials on intensity-modulated radiotherapy in head and neck cancer and 3D radiation in prostate cancer have been landmark studies.^9,10^ The UK has come late to the proton game, yet is not so far behind. It will likely have contributions to make in physics, engineering, and biology, but its true strength will be in the area of comparative effectiveness. Exactly how much better is proton therapy, if at all, and for which cancers and in which parts of the body? This is the data that will inform payers and policy makers around the globe, and determine future clinical applications. The UK has a huge contribution yet to make.
